# Case report of disseminated borrelial lymphocytoma with isolation of *Borrelia burgdorferi* sensu stricto in chronic lymphatic leukemia stage Binet A—an 11 year follow up

**DOI:** 10.3389/fmed.2024.1465630

**Published:** 2024-10-18

**Authors:** Heidelore Hofmann, Gabriele Margos, Antonia Todorova, Ingo Ringshausen, Konstantin Kuleshov, Volker Fingerle

**Affiliations:** ^1^Department of Dermatology and Allergy, University Hospital Rechts der Isar, Technische Universität München, Munich, Germany; ^2^National Reference Center for Borrelia, Bavarian Health and Food Safety Authority, Oberschleissheim, Germany; ^3^Department of Public Health, City of Munich, Munich, Germany; ^4^III Medical Department for Hematology and Hematooncology, University Hospital Rechts der Isar, Technische Universität München, Munich, Germany; ^5^University College London Cancer Institute, London, United Kingdom; ^6^Central Research Institute of Epidemiology, Moscow, Russia

**Keywords:** disseminated *Borrelia* lymphocytoma, skin infiltrates, leukemia cutis, chronic lymphoproliferative disease, case report, Lyme borreliosis, *Borrelia burgdorferi*

## Abstract

We report a rare manifestation of cutaneous borreliosis in a patient with pre-existing malignant lymphoproliferative disease, in particular chronic lymphocytic B cell leukemia (B-CLL). The patient’s cutaneous lesions were initially diagnosed histologically as leukemia cutis. Distribution pattern of the skin lesions were in typical localizations for borrelial lymphocytoma. *Borrelia burgdorferi* sensu stricto was isolated and cultured from two sites (ear, mammilla). Antibiotic therapy improved the cutaneous lesions and the general condition of the patient. However, a second round of antibiotic therapy was required to resolve the lesions. At eleven years of follow-up the patient’s skin was clear and she still had a stable condition of B-CLL without chemotherapy. In conclusion, the patient suffered from Lyme borreliosis (*Borrelia* lymphocytoma) and the cutaneous symptoms were aggravated by the underlying condition of chronic B-CLL condition.

## 1 Introduction

The association of certain types of cancer with bacterial, viral or protozoan infections is well established (1). Although globally the vast majority of cancers are attributable to viral or *Helicobacter* infections (1), infections with *Borrelia burgdorferi* have also been associated with lymphoma (2) and chronic lymphatic B cell leukemia (B-CLL) (3–6). In about 4–25% of patients with B-CLL, cutaneous infiltration of monomorphous lymphocytes may point to an unfavorable progression and can lead to the condition of leukemia cutis (LC) (7). In LC the appearance of skin lesions is diverse and can present as localized or generalized papules, plaques, nodules or tumors [reviewed by Cho-Vega et al., Cerroni et al., and Robak and Robak (8–10)]. Morphological pattern of cell infiltration may be perivascular, nodular and diffuse or band-like. Immunohistologically, the phenotype displayed by these monotonous small lymphocytes has been described as CD20+/CD43+ (4); CD20+, CD5+, CD43+; CD19+/CD5+ (9); CD5+/CD20+ (5); CD19+/CD20 ± (weak)/CD5+/CDc23+ (6). Nonspecific changes of the skin may also be associated with infectious agents (7). In the case described here the cutaneous lesions resembled borrelial lymphocytoma. Several publications reported such skin lesions in association with suspected *B. burgdorferi* infections at sites typical for *B. burgdorferi*-associated lymphocytoma (e.g., nipple, ear) or erythema migrans (4, 5). Kempf and colleagues (6) reported the unusual case of cutaneous Lyme Borreliosis in a patient with B-CLL where a T-cell rich infiltrate predominated. Dermatological examination, antibody laboratory tests, isolation of *Borrelia* and molecular evidence supported the diagnosis of Lyme borreliosis.

To date, the association of primary cutaneous lesions and *Borrelia* has been established from DNA evidence of the infectious agent (4–6). Here, we report isolation and *in vitro* cultivation of *B. burgdorferi* sensu stricto from a patient with chronic lymphatic B-cell leukemia (B-CLL). Due to the diagnosis of leukemia cutis, chemotherapy had been recommended but *B. burgdorferi* s.s. could be isolated from the skin lesions and the skin manifestations were cleared after antibiotic therapy, providing strong evidence for a cutaneous infection by *B. burgdorferi* s.s.

## 2 Case description

**Figure 1:**
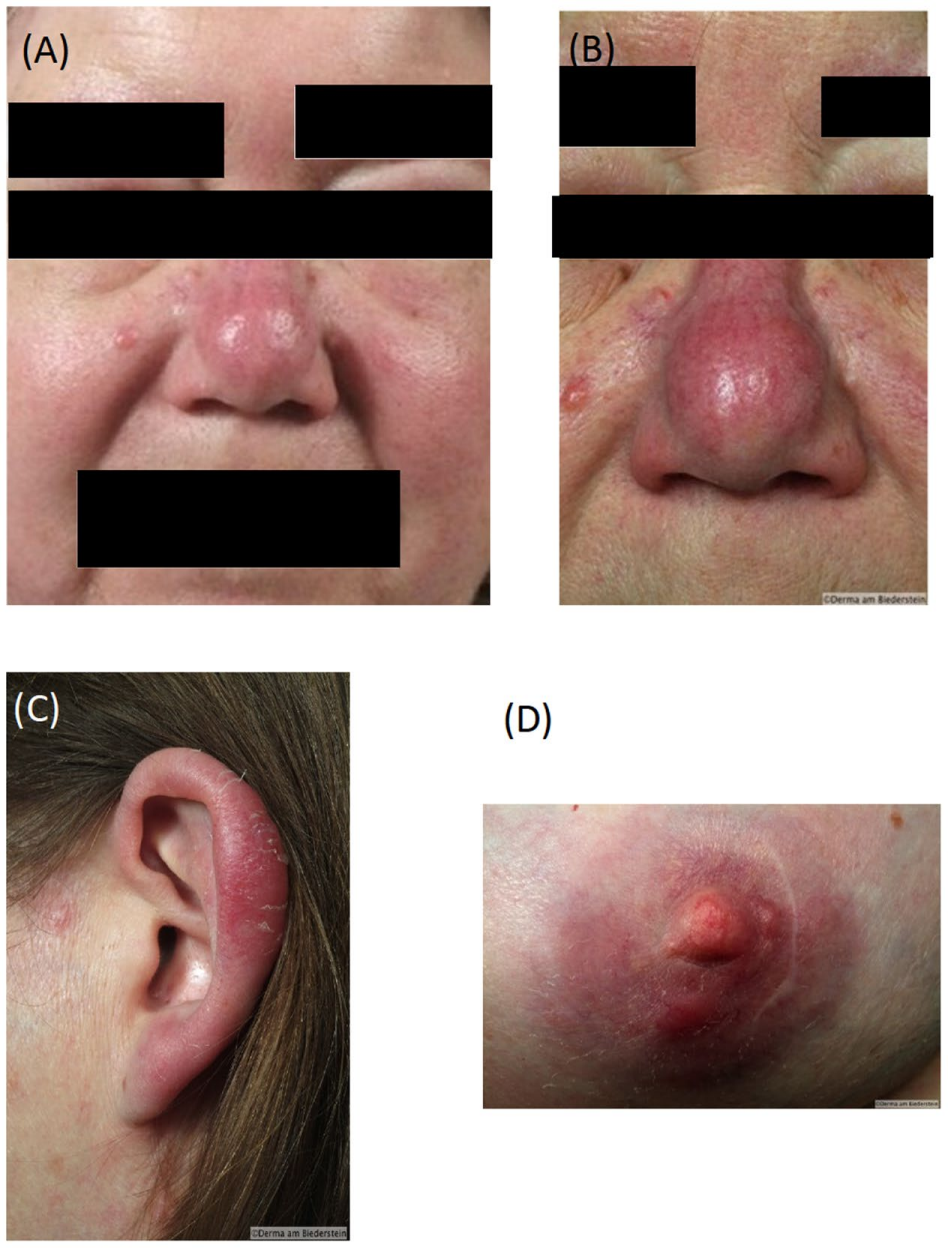
Diffuse oedema and infiltration of the face at presentation **(A)**, Erythematous nodular skin infiltrates typical borrelial localization bevor therapy: **(B)** nose, **(C)**, ear **(D)** nipple.

A 58-year old female patient with known chronic lymphatic B cell leukemia (B-CLL) (2) in stable disease for five years developed diffuse erythematous edema and infiltrations of the face with nodular lesions on nose, left ear and both nipples (Figures 1A–D) suggesting leukemic infiltrates by hematologists. Upon clinical dermatological examination, skin biopsies were taken from the affected areas and evaluated by two independent histopathologists. The findings corresponded with cutaneous leukemic infiltrates of the underlying B-CLL. lmmunohistochemical antigen staining of the lymphocytic infiltrates gave positive results for CD20, CD3, CD79a, CD25 and negative for CD5 and myeloperoxidase (MPO). Immunohistological examination of the bone marrow showed 60% infiltration of neoplastic cells with expression of CD20, CD5 and CD23 and a proliferation rate of 10%.

**Figure 2:**
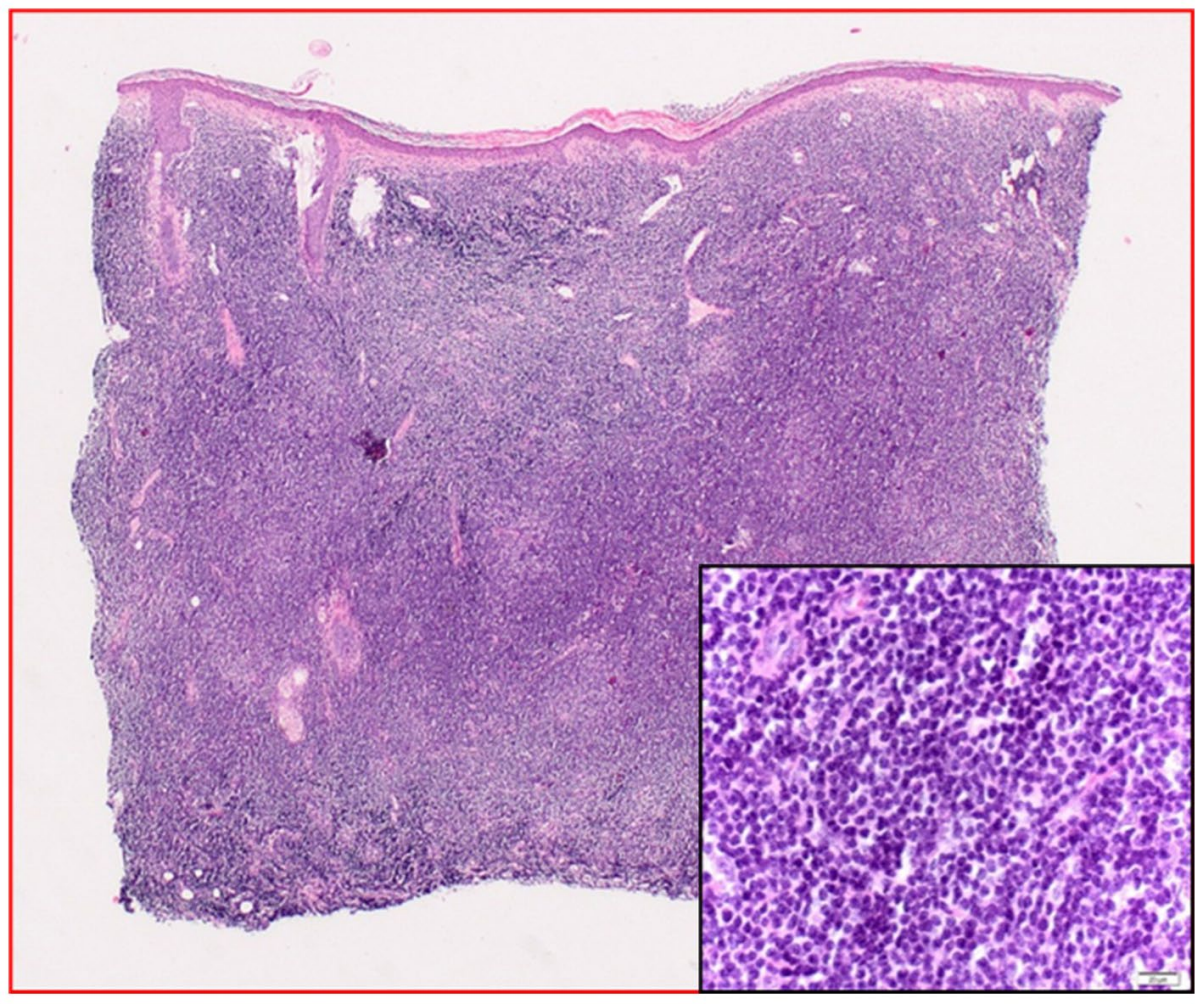
Histopathology (hematoxylin–eosin staining) before treatment: dense cellular infiltrates of the entire dermis with monomorphous plasmocytoid cells. The inlet is an enlargement showing a close-up of the plasmocytoid cells.

Based on the diagnosis of leukemia cutis chemotherapy was recommended. Due to the absence of CD5 and MPO staining, before initiation of the therapy the patient presented for a second assessment at the Dermatology Department, Klinikum rechts der Isar, Technical University Munich, Germany. The affected areas strongly resembled a distribution pattern typical for borrelial lymphocytoma. *Borrelia* serology by ELISA (Sonicate IgG und IgM Elisa; Virotech, Germany) showed high concentrations of anti-borrelial IgG antibodies and in immunoblot (Borrelia ViraStripe® IgG and Borrelia ViraStripe® IgM, Viramed, Germany) a broad spectrum of *B. burgdorferi* specific bands were visible (Table 1), which is consistent with late Lyme borreliosis. The patient had not previously received a diagnosis and treatment for Lyme borreliosis. Moreover, she reported increased fatigue and impairment in performing her daily activities. We diagnosed a disseminated borrelial lymphocytoma. Skin biopsies from the left ear and from the right mammilla were evaluated histopathologically (Figure 2) and using immunostaining (not shown). Microbiologically, *B. burgdorferi* DNA was detected by PCR in the affected lesions; moreover, *B. burgdorferi* s.s. was successfully cultured from both locations. These isolates were designated PFhe_I and PFhe_II.

**Table 1:** Chronological decrease of borrelial antibodies in ELISA and immunoblot pre- and post-therapy.

Date	Sonicate IgM (VE: 9–11)	Immunoblot IgM	Sonicate IgG (VE: 9–11)	Immunoblot IgG
11.05.10	5.13	Negative	57.07	P83, p58, p41, p39, p21, DpbA, VIsE (p43, p30, OspC) kD
07.07.10	1.58	Negative	62.5	P83, p58, p41, p39, p21, DbpA, VIsE (p43) kD
15.09.10	1,57	Negative	59.34	P83, p58, p43, p41, p39, p21, DbpA, VIsE (OspC) kD
06.07.11	0.95	Negative	44.01	P83, p41, p39, p21, DbpA, VIsE (p58, p43) kD
01.02.12	1.07	Negative	43.66	P41, p39, p21, DbpA, VIsE (p83, p58) kD
14.11.12	0.33	Negative	47.87	P41, DbpA, VIsE (p39) kD
27.02.23	0.22	Negative	8.58	VlsE

Systemic intravenous antibiotic treatment with Ceftriaxone 2 g per day was performed over 21 days. During the therapy significant regression of the erythematous infiltrates on the ears, nose and on the mammillae and reduction of the periorbital edema was noticed (Supplementary Figures S1A–C). The general condition of the patient rapidly improved.

Three months after antibiotic therapy skin biopsies were taken from the mammilla and from the ear. Histopathological assessment showed lymphocytic infiltrations and para-immunoblastic cells. Immunohistochemical analysis showed positive cell staining for antigens CD20, CD3, CD5 and CD23 but negative staining for CD43 with a cell proliferation index of 5%. Although positive cell staining with these immunological markers may be consistent with the diagnosis of leukemia cutis, a control PCR using *B. burgdorferi* specific primer on material from the left ear performed four months after Ceftriaxone therapy resulted in a positive PCR, indicating the persistent presence of *B. burgdorferi* DNA.

A second course of treatment with Ceftriaxone 2 g over 21 days was initiated. Following this second antibiotic treatment, PCR analyses of DNA isolated from tissues of the ear lobe and mammilla gave negative results for *Borrelia* DNA. *Borrelia* antibodies decreased slowly over two years. At the end of the second treatment complete regression of the skin infiltrates was observed. Histopathological examination of the skin 17 months after Ceftriaxone treatment showed mixed T- and B-cell lymphocytic infiltration (Supplementary Figure S2) with CD3 and CD20 positive and CD30 negative cells in a ratio of 1:1 without signs of malignancy.

Intriguingly, the patient underwent a significant hematological remission following antibiotic treatment, suggesting that the *Borrelia* infection caused a flair of the underlying CLL. Subsequently, the patient remained under hematological control and the chronic lymphatic leukemia remained stable (65,000 leucocytes, stage Binet A) without requirement for initiating treatment. A follow up examination after 11 years showed a complete clearance of all cutaneous infiltrates (Supplementary Figures S3A–C) and a good general condition. Serologic examination by ELISA and immunoblot could only detect IgG antibodies against VlsE (Table 1). A timeline is given in Supplementary Figure S4.

## 3 Genome analyses of isolates PFhe_I and PFhe_II

To establish whether the infecting strains of *B. burgdorferi* s.s. PFhe_I and PFhe_II have virulence determinants that may explain the fulminant course of symptoms observed in the present study, we analyzed the genome of the isolates obtained from the patient. We sequenced the genome of PFhe_I using Illumina and Pacific Bioscience single-molecule sequencing in real-time (SMRT) technology and genomes of several European isolates (Multilocus sequence typing (MLST) sequence types (ST) ST20, ST21, ST24, ST284) using Illumina MiSeq technology (see Supplementary Table S1 for details). SMRT sequences were assembled at the Norwegian Genome Sequencing Center as part of the sequencing contract. Illumina sequences were assembled using SPAdes v. 3.11.1 (11).

The genome of PFhe_I consists of a linear chromosome (910 kbp), nine linear and five circular plasmids (Supplementary Table S2) which is in the normal range of plasmid numbers in *B. burgdorferi* s.s. (12). The presence and identity of plasmids was confirmed using PFam32 sequences (13). The genome analysis further revealed that the genomes of PFhe_I and PFhe_II were highly similar.

In previous studies *B. burgdorferi* s.s. isolates PFhe_I and PFhe_II have been determined to belong to ST21 (14); this ST group also includes patient isolates such as PG_I (patient’s symptoms: neuroborreliosis) and PSst (patient’s symptoms: acrodermatitis chronicum atrophicans (ACA)) (see Supplementary Table S1). Phylogenetic studies using MLST sequences (15) and whole genome data (14, 16) showed that other European isolates of *B. burgdorferi* s.s. belonging to ST20 and ST284 formed closely related cluster while others, i.e., ST24, were more distantly related (16).

Genome analyses were conducted using Spine and ClustAGE (17). These software tools identify regions in the core and accessory genomes of groups of bacteria that may be involved in pathogenicity or adaptation to specific niches. The accessory genomes were binned (clustered) according to homology and their distribution in the studied isolates is shown in Supplementary Figure S5. In total, there were 118 clusters of homology in the accessory genome of the 12 isolates. When using a similarity threshold of 85%, a 2 kb region (bin27) was absent in the genomes of PFhe_I and PFhe_II. Reducing the similarity threshold to 70%, this region matched locations on different cp32 plasmids in PFhe_I and included a coding sequence for a plasmid partitioning gene (data not shown). A unique 215 bp long sequence (bin115) was found in a repetitive region exclusively in the genome of PFhe_I.

We also created a neighbor joining tree based on the Bray–Curtis dissimilarity matrix from distributions of accessory genome elements (17, 18). This statistical approach is used to quantify the accessory genome similarity of isolates. The resulting tree visualizes clustering of strains according to the similarity of accessory elements. It is obvious that the accessory elements of the PFhe_I and PFhe_II genomes clearly form a unique cluster among all isolates (Supplementary Figure S6).

Outer surface proteins (Osp) of *Borrelia* may act as virulence determinants (19). A variable and highly immunogenic outer surface protein of *Borrelia* is the OspC (20). This molecule is important for host invasion and/or tick salivary gland invasion (21, 22). In North America, the protein has been associated with invasiveness in Lyme borreliosis and five *ospC* major groups appear to be often involved in disseminated disease (23, 24). Major groups of *ospC* are determined by sequence divergence of >8%. More than 25 *ospC* major groups have been determined in North America (20, 25) but the *ospC* types of European isolates have not been determined in detail. To determine the *ospC* major group of the isolates investigated here, we downloaded from GenBank the sequences investigated by Travinsky et al. (25) and generated a phylogenetic network using Splitstree (26) (Supplementary Figure S7). This analysis revealed that the *ospC* of ST21 (which included PFhe_I, PFhe_II, PG_I and PSst) were identical but did not cluster with any of the known *ospC* major groups, thus representing a novel *ospC* major group. ST20 isolates clustered with *ospC* major group B, ST24 isolates clustered with *ospC* major group L while ST284 also possessed a new *ospC* major group (Supplementary Figure S7).

Except for the unique low complexity region (bin115, Supplementary Figure S5), absence of a 2 kb sequence located in plasmid cp32 in other isolates, and a new *ospC* major type (Supplementary Figure S7), our genome analyses did not reveal more substantial genomic differences between PFhe and other *B. burgdorferi* s.s. isolates originating from patients with Lyme borreliosis manifestations such as neuroborreliosis or ACA.

## 4 Discussion

We report a rare manifestation of disseminated cutaneous borreliosis in a patient with pre-existent malignant lymphproliferative disease, chronic lymphatic leukemia (B-CLL) Binet A. Distribution pattern of the skin lesions showed symmetric, disseminated infiltrates in typical localizations for borrelial lymphocytoma, although in unusual severe manifestation. Initially, cutaneous infiltrates were diagnosed histologically as leukemia cutis although, contrary to previous publications, CD5 stained negative in skin infiltrates. This discrepancy and the resemblance of skin lesion to borrelial lymphocytoma prompted the dermatological assessment in which titers and spectrum of anti-borrelial antibodies suggested late Lyme borreliosis (27). Isolation of live *B. burgdorferi* s.s. MLST ST21 from two sites (ear, mammilla) provided further evidence that the *Borrelia* infection was the cause of the skin lesions. In line with this, antibiotic therapy with Ceftriaxone for Lyme Borreliosis resolved the cutaneous lesions and improved the general condition of the patient. However, after the first antibiotic treatment borrelial DNA persisted in the affected lesions for several months. Immunohistology now showed a cell staining positive for CD20, CD3, CD5 and CD23 consistent with leukemia cutis (8, 9). Although *B. burgdorferi* could not be cultivated after the initial antibiotic therapy the persistence of DNA may have been a trigger for perpetuation of lymphoproliferation. A second round of antibiotic therapy resolved the residual skin infiltrates and borrelial DNA was no longer detectable.

We used whole genome sequencing to show that the genomes of PFhe_I and PFhe_II were highly similar (chromosome, cp26, lp17, lp25, lp28-2, lp28-4, lp28-7, lp28-9, lp36, lp54 > 99^.^5%). Phylogenetic clustering of accessory genome elements identified only small differences between PFhe_I/PFhe_II and the other *B. burgdorferi* s.s. isolates that were isolated from patients with different LB symptoms such as neuroborreliosis or ACA. This indicates (i) that these two isolates have a common genetic background with other isolates, and (ii) that the observed clinical symptoms may due to other factors than isolate-specific genome elements. Sequence analysis of *ospC* revealed that the *ospC* gene of PFhe constitutes a novel major group. Although OspC is a known virulence determinant of *B. burgdorferi* s.s. (19), the molecule on its own is insufficient to change the dissemination type of an isolate as shown by *ospC* replacement experiments (28). Thus, it will require further investigations to see if there is clinical significance in the novel *ospC* gene.

Association of Lyme borreliosis and lymphoproliferative diseases has been reported (4–6, 29). Moreover, infectious agents have been suggested as trigger for the development of cutaneous infiltrates in patients with malignant lymphoproliferative diseases (3, 4). We suggest that the chronic functional deficiency of B- and T-cell interaction in chronic B-cell leukemia has triggered the fulminant course of the cutaneous borrelial infection. Remarkably, at eleven years of follow-up the patient still has a stable condition of B-CLL stage Binet A. In the case of our patient skin infiltrates completely disappeared after Ceftriaxone therapy and new skin infiltrates have not been observed. Thus, antimicrobial treatment should be part of the first line therapy in patients with lymphoproliferative diseases and positive findings in borrelial diagnostics.

Taken together, our study shows that aggravation of clinical symptoms occurred in a patient with lymphproliferative disease following *Borrelia* infection. Several rounds of antibiotic treatment resolved the *Borrelia* infection. The patient’s condition has remained stable for 11 years. We conclude that the chronic B-CLL condition of the patent has aggravated the cutaneous symptoms of borrelial infection.

## Data Availability

The datasets presented in this study can be found in online repositories. The names of the repository/repositories and accession number(s) can be found in the article/Supplementary material.
