# Supplementary material for: Case report of disseminated borrelial lymphocytoma with isolation of Borrelia burgdorferi sensu stricto in chronic lymphatic leukemia stage Binet A—an 11 year follow up
Source: Front Med (Lausanne). 2024 Oct 18;11:1465630. doi: 10.3389/fmed.2024.1465630 (PMC11527655; doi:10.3389/fmed.2024.1465630)
Supplement: Supplementary file 1 [file Data_Sheet_1.pdf]

Supplementary Material

1 Supplementary Figures and Tables

Supplementary Figure S1. Regression of cutaneous infiltrates after systemic antibiotic treatment: (A) face, (B) ear, (C) nipple

Supplementary Figure S2. Histopathology of the skin after 17 months after antibiotic treatment showing mixed T- and B-cell lymphocytic infiltration without signs of malignancy (see inlet for detailed view).

Supplementary Figure S3. Regression of cutaneous infiltrates 11 years after systemic antibiotic treatment: (A) face, (B) ear, (C) nipple

Supplementary Figure S4. Timeline of course of symptoms, treatment including laboratory test results and follow-up

Supplementary Figure S5. Visual representation of bins of accessory genome of 12 genomes of *B. burgdorferi* sensu stricto strains

Supplementary Figure S6. Bray-Curtis distance matrix based on the distributions of accessory elements

Supplementary Figure S7. A neighbor network using known *ospC* major groups

Supplementary Table S1. Isolates used for genome comparison with PFhe_I

Supplementary Table S2. Genome content of PFhe_I

References

1.1 Supplementary Figures

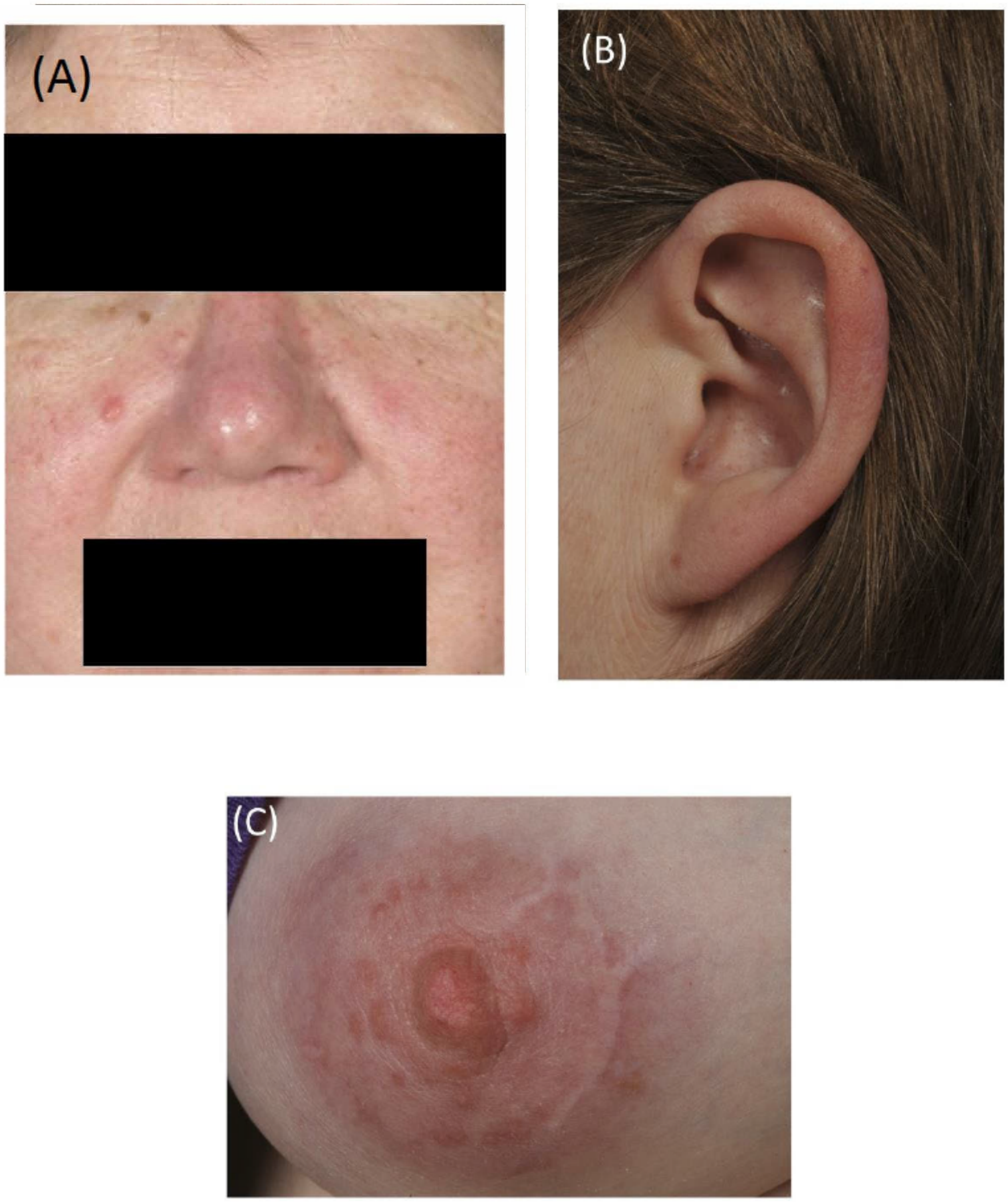

Supplementary Figure S1. Regression of cutaneous infiltrates after systemic antibiotic treatment: (A) face, (B) ear, (C) nipple

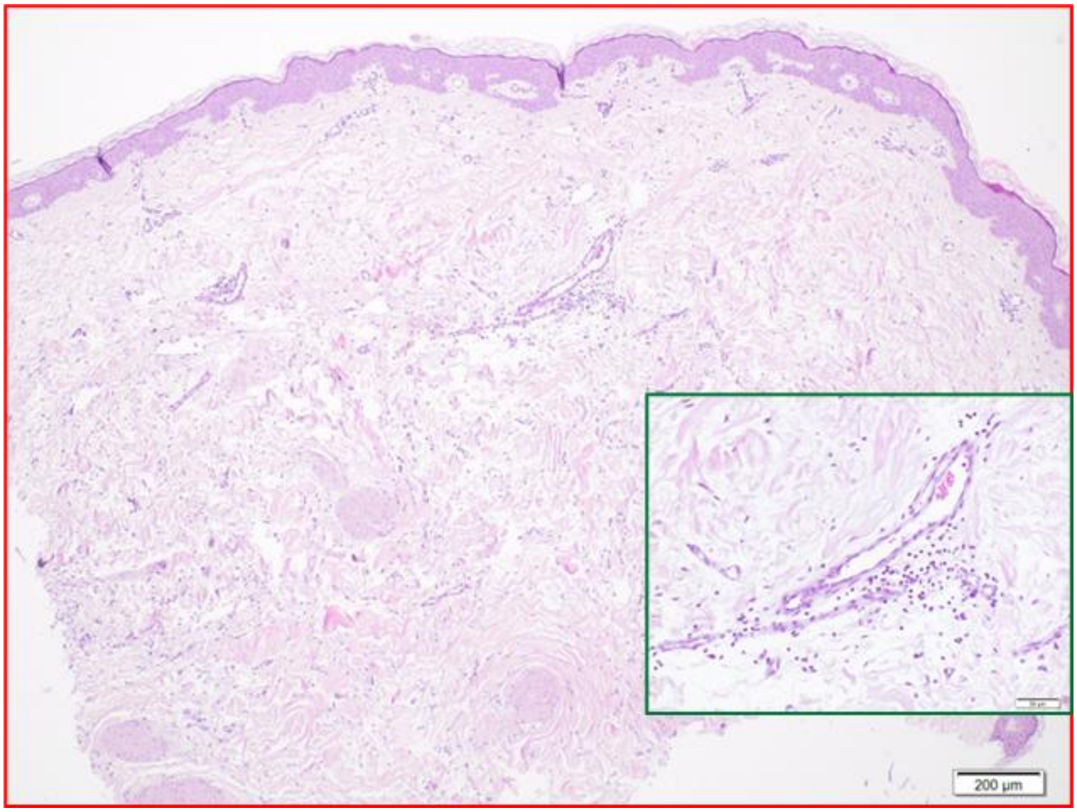

Supplementary Figure S2. Histopathology of the skin 17 months (stained with hematoxylin-eosin) after antibiotic treatment showing mixed T- and B-cell lymphocytic infiltration without signs of malignancy (see inlet for detailed view).

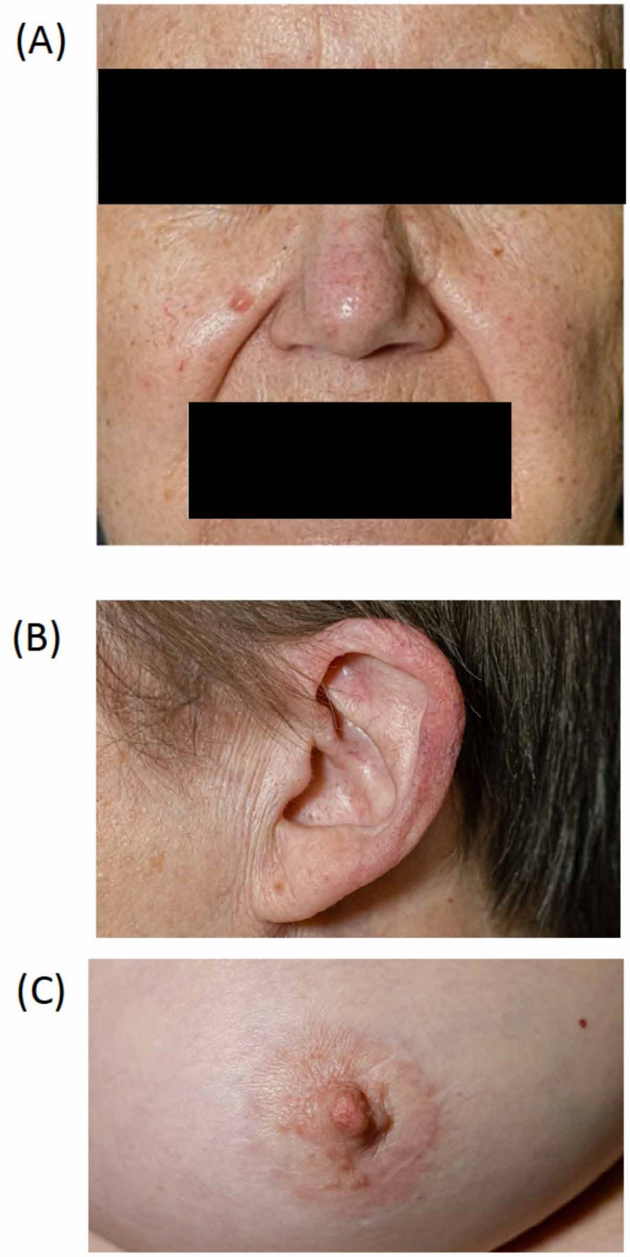

Supplementary Figure S3. Regression of cutaneous infiltrates 11 years after systemic antibiotic treatment: (A) face, (B) ear, (C) nipple

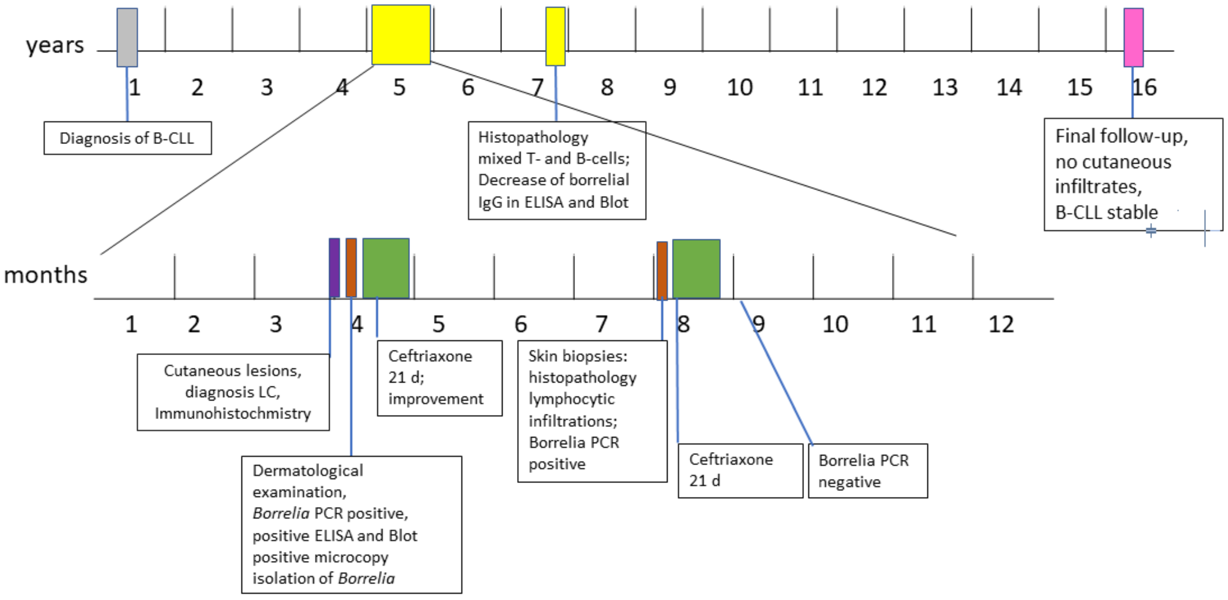

Supplementary Figure S4. Timeline of course of symptoms, treatment including laboratory test results and follow-up

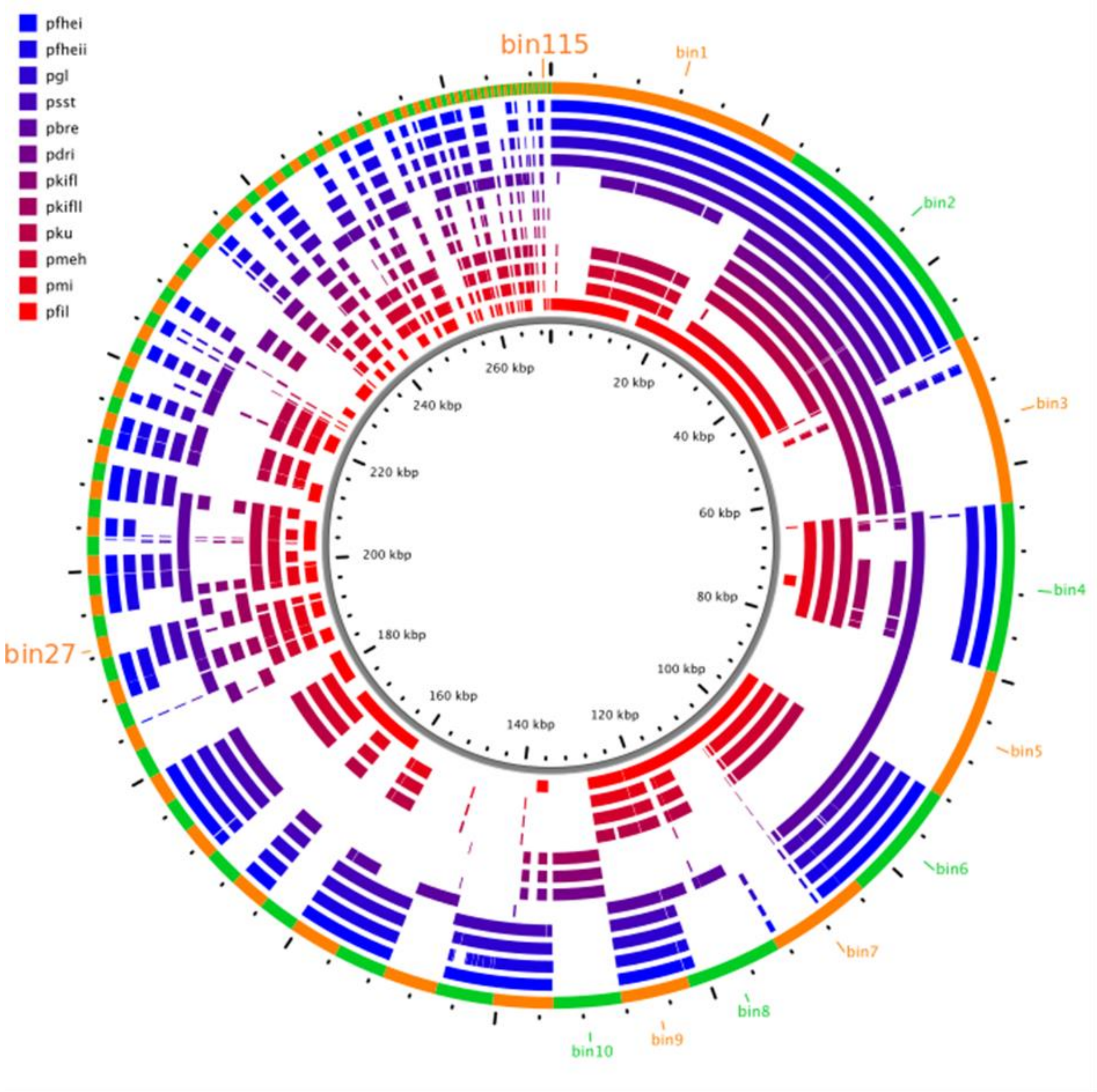

Supplementary Figure S5. Visual representation of bins of accessory genome of 12 genomes of *B. burgdorferi* sensu stricto strains generated using Spine v.0.3.1 and ClustAGE v.0.8 (1, 2). See Table S1 for strains included.

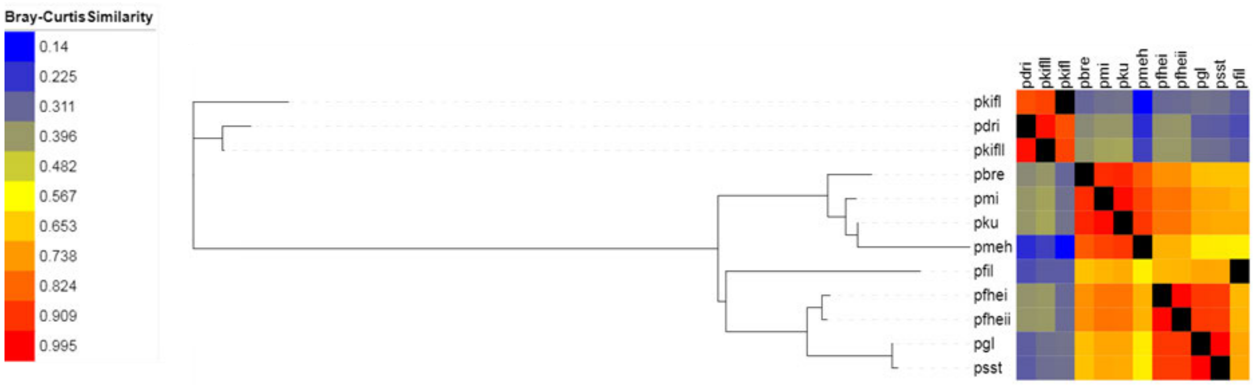

Supplementary Figure S6. Bray-Curtis distance matrix (3) based on the distributions of accessory elements that are used to create a neighbor joining tree of accessory element distribution patterns of 12 genomes of *B. burgdorferi* ss isolates. See Table S1 for isolates included

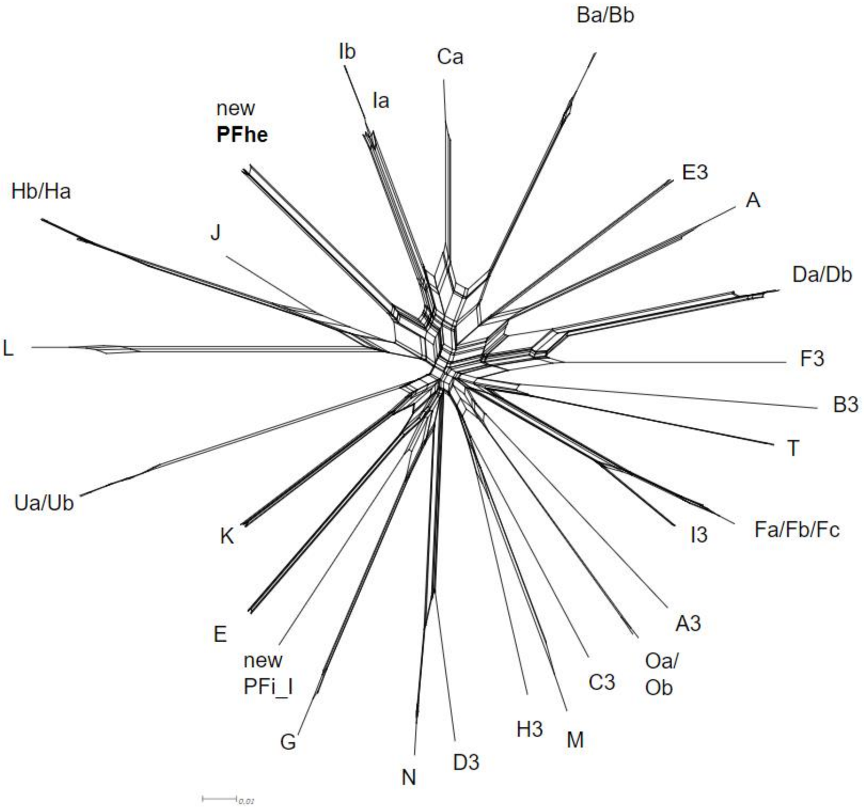

Supplementary Figure S7. A neighbor network using known ospC major groups (4) was generated using Splitstree (5). OspC major groups are designated with letters. European isolates with ospC major groups that did not cluster with any previously characterized groups are designated “new”. All isolates that were defined as ST21 (PFhe, PG_I, PSst) possessed the same ospC. PFi_I (ST284) also had a new ospC major group.

Supplementary Table S1. Isolates used for genome comparison with PFhe_I; symptoms of patient from which the isolates were obtained; Multilocus sequence typing (MLST) sequence type (ST); data available for genome analysis GenBank SRA Bioproject PRJNA2770494

**Isolate name**	**symptoms**	**MLST ST**	**Reads available**	**GenBank accession numbers**
**PFhe_I**	BLZ	ST21	PacBio/TS/MP	SAMN03269138
PFhe_II	BLZ	ST21	TS/MP	SAMN03269139
PG_l	NB	ST21	Nextera	SAMN03269141
PSst	ACA	ST21	Nextera	SAMN03269149
PBre	EM	ST20	TS/MP	SAMN03269136
PKu	NB	ST20	Nextera	SAMN03269145
PMeh	A	ST20	TS	SAMN03269147
PMi	NB	ST20	Nextera	SAMN03269148
PDri	EM	ST24	Nextera	SAMN03269137
PKif_I	ND	ST24	Nextera	SAMN03269143
PKif_II	ND	ST24	Nextera	SAMN03269144
PFi_I	EM/ACA	ST284	TS	SAMN03269140

Symptoms
A = arthritis
ACA= acrodermatitis chronicum atrophicans
BLZ = B-cell lymphocytome
EM = erythema migrans
NB = neuroborreliosis
ND=no data

Reads available
Illumina MiSeq reads
Nextera = Nextera library construction
TS = TrueSeq library construction
MP= Mate paired library construction (3000-5000bp)

Supplementray Table S2. Genome content of PFhe_I as determined by Pacific BioScience SMRT technology

**Genome element (PFam32 match)**	**Size (bp)**
chromosome	910254
lp17	17337
lp25	24445
lp28-2	30086
lp28-3	30430
lp28-4	28903
lp28-7	15159
lp28-9	28224
lp36	36948
lp54	53780
cp26	26503
cp32-7	31008
cp32-6+10	47043
cp32-5	30705
cp32-12	30708

References

1. Ozer EA. ClustAGE: a tool for clustering and distribution analysis of bacterial accessory genomic elements. BMC Bioinformatics. 2018;19(1):150.

2. Ozer EA, Allen JP, Hauser AR. Characterization of the core and accessory genomes of *Pseudomonas aeruginosa* using bioinformatic tools Spine and AGEnt. BMC Genomics. 2014;15(1):737.

3. Shapiro BJ, Friedman J, Cordero OX, Preheim SP, Timberlake SC, Szabo G, et al. Population genomics of early events in the ecological differentiation of bacteria. Science. 2012;336(6077):48-51.

4. Barbour AG, Travinsky B. Evolution and Distribution of the *ospC* Gene, a Transferable Serotype Determinant of *Borrelia burgdorferi*. MBio. 2010;1(4).

5. Huson DH. SplitsTree: analyzing and visualizing evolutionary data. Bioinformatics. 1998;14(1):68-73.
